# Green Tea Polyphenols Protect against Acetaminophen-Induced Liver Injury by Regulating the Drug Metabolizing Enzymes and Transporters

**DOI:** 10.1155/2020/2696432

**Published:** 2020-11-19

**Authors:** Le Lv, Chenshu Xu, Xiaoye Mo, Hai-Yan Sun, Huichang Bi

**Affiliations:** ^1^Guangdong Provincial Key Laboratory of New Drug Design and Evaluation, School of Pharmaceutical Sciences, Sun Yat-Sen University, Guangzhou 510006, China; ^2^Postdoctoral Innovation Practice Base, Shenzhen Polytechnic, Shenzhen 518055, China; ^3^School of Pharmaceutical Sciences, Health Science Center, Shenzhen University, Shenzhen 518060, China; ^4^Institute of Marine Biomedicine, Shenzhen Polytechnic, Shenzhen 518055, China

## Abstract

Green tea polyphenols (GTPs) have been shown to exhibit diverse beneficial effects against a variety of diseases. Acetaminophen (APAP) overdose is one of the most frequent causes of drug-induced liver injury. In the current study, we aimed to investigate the protective effect of GTP on APAP-induced liver injury in mice and the underlying mechanisms involved. Male C57BL/6J mice were treated orally with different doses of GTP (37.5, 75, or 150 mg/kg) 4 h after APAP overdose (400 mg/kg) and continuously given every 8 h until sacrificed at 4, 12, 20, and 48 h after the first treatment of GTP. Survival rate and histological and biochemical assessments were performed to evaluate the APAP-induced liver injury. Protein expression of multiple drug metabolizing enzymes and transporters was measured to demonstrate the possible mechanisms involved. Our results revealed that administration of different doses of GTP significantly alleviated APAP-induced liver injury by improving the survival rate, hepatocellular necrosis, and ALT/AST/GSH levels after APAP overdose (400 mg/kg). The protein expression of APAP-induced drug transporters and metabolizing enzymes was mostly induced by GTP treatment, which was followed by reduction in drug transporters at the later time points. The current study collectively demonstrated that GTP protects against APAP-induced liver injury, possibly through regulating drug metabolizing enzymes and transporters after APAP overdose.

## 1. Introduction

Drug-induced liver injury has been identified as an important clinical issue which can be caused by various physician-prescribed medications, over-the-counter (OTC) medications, herbal medicines, and vitamin supplements [1]. Acetaminophen (APAP) is a widely prescribed analgesic and antipyretic drug, which is relatively safe and effective when administered at therapeutic doses. Under normal condition, about 85% of APAP undergoes phase II conjugation and converts into nontoxic sulfated or glucuronidated metabolites by UDP-glucuronosyltransferases (UGTs) or sulfotransferases (SULTs), respectively. However, the overdose of APAP leads to acute liver failure, which remains as the major cause of drug-induced liver injury. It has been well established that metabolic activation of APAP to N-acetyl-p-benzoquinone imine (NAPQI), a toxic and highly reactive intermediate, by multiple cytochrome P450 enzymes serves as the critical step initiating the hepatotoxicity. The increasing amount of NAPQI depletes liver glutathione (GSH) and covalently binds to cellular proteins and DNA, which eventually results in oxidative stress, mitochondrial damage, and hepatocellular necrosis [2–4]. Increasing the expression of membrane transporters such as P-glycoprotein (P-gp) and multidrug resistance-associated proteins (Mrps) has been shown to facilitate the excretion of glucuronate and sulfate, and GSH conjugates from the liver [5].

Identifying therapeutically effective components from natural products proves to be a promising way to ameliorate APAP overdose. For example, schisandrol B, an important active component isolated from *Schisandra sphenanthera*, protects against APAP-induced hepatotoxicity [6]. Tea, made from the leaves of *Camellia sinensis* L., is one of the most popular beverages in the world. Depending on the different tea processing, the final products are mainly classified into several varieties, among which green tea accounts for a large portion of total tea consumption. Longjing green tea, also known as Dragon Well green tea, is a famous variety of green tea mainly grown near the city of Hangzhou in China for more than one thousand years. Green tea is a major source of dietary polyphenols and has been studied for its multiple pharmacological and physiological effects for a long time, including inhibition of carcinogenesis and antioxidative and anti-inflammatory activities [7]. Previous study has revealed that epigallocatechin-3-gallate (EGCG), one of the major catechins, identified in green tea reduces hepatic oxidative stress and lowers CYP-mediated bioactivation and toxicity of acetaminophen in rats [8]. The aim of the current study, however, was to identify the protective effects of GTP against APAP-induced liver injury in APAP overdose mice. Furthermore, the possible underlying mechanisms and the time course involving regulating the drug metabolizing enzymes and transporters were also investigated.

## 2. Materials and Methods

### 2.1. Chemicals and Reagents

Longjing green tea was purchased from Yipinxuan Teahouse (Shenzhen, Guangdong, China). APAP was purchased from MCE Co. (China). The anti-mrp2, anti-cyp2e1, anti-cyp1a2, anti-cyp3a4, anti-P-gp, anti-ugt1a6, and anti-gapdh antibodies and goat anti-rabbit immunoglobulin G (IgG) were obtained from Abcam (Cambridge, UK). The anti-sult1a1 antibody was obtained from Bioss (Beijing, China). The gallic acid (GA), gall catechin (GC), epigallocatechin (EGC), catechin (C), caffeine (CAF), epicatechin (EC), epigallocatechin gallate (EGCG), gallate (GCG), epicatechin gallate (ECG), catechin gallate (CG), theaflavin (TF), theaflavin-3-gallate (TF-3-G), and theaflavin-3′-gallate (TF-3′-G) standards were all of chromatographic grade and purchased from Sigma-Aldrich (St. Louis, MO, USA).

### 2.2. Preparation of Green Tea Polyphenol Extract (GTP)

The extraction of green tea polyphenols was conducted according to the Chinese national standard GB/T8313-2018 and modified accordingly. The green tea powder (20 g) was soaked with 500 mL preheated 70% methanol ((*w*/*v*) = 1 : 25) and extracted in 70°C waterbath for three times. The crude extract was combined, filtrated, evaporated, extracted with chloroform ((*v*/*v*) = 3 : 1), condensed to 5% of the original volume, and dissolved in ultra-pure water. The water soluble extract was freeze-dried and stored at room temperature in a light-proof condition.

### 2.3. Development of an HPLC Method for Determination of GTP

Five mg of gallic acid (GA), gall catechin (GC), epigallocatechin (EGC), catechin (C), caffeine (CAF), epicatechin (EC), epigallocatechin gallate (EGCG), gallocatechin gallate (GCG), epicatechin gallate (ECG), catechin gallate (CG), theaflavin (TF), theaflavin-3-gallate (TF-3-G), and theaflavin-3′-gallate (TF-3′-G) was, respectively, dissolved in 5 mL of 50% methanol as the standard solution and stored in −80°C for further HPLC analysis. The HPLC separation was achieved by using a C18 column (Agilent ZORBAX Eclipse Plus Phenyl-Hexyl, 4.6 mm × 250 mm, 5 *μ*m) at 40°C. The mobile phase consisted of 0.15% acetic acid (A)−100% acetonitrile (B) and programmed by 98-97% (*v*/*v*) A at 0–10 min, 97-93% A at 10–15 min, 93-90% A at 15–30 min, 90–88% A at 30–55 min, 88-86% A at 55–65 min, 86-85% A at 65–75 min, 85–75% A at 75–78 min, 75-70% A at 78–90 min, and 70–98% A at 90–100 min. Detection was performed at a wavelength of 280 nm with a flow rate of 1 mL/min. The total running time was 100 min for each sample.

### 2.4. Animals

Male C57BL/6J mice (6–8 weeks old) were supplied by Laboratory Animal Center of Southern Medical University (Guangzhou, China). Animal license number is SCXK (Guangdong) 2016-0041. All animals were acclimatized to the laboratory environment for 2 weeks before the experiment. The animals were maintained under controlled conditions (23 ± 1°C, 55 ± 5% humidity and 12 h light-dark cycle) with free access to standard rodent chow and water. All procedures were in accordance with the Regulations of Experimental Animal Administration issued by the Ministry of Science and Technology of the People's Republic of China (http://www.most.gov.cn).

Acetaminophen was suspended in 1% CMC-Na before use. Mice were randomly divided into five groups with 10 mice in each group: (1) control vehicle, (2) APAP (400 mg/kg) + vehicle, (3) APAP (400 mg/kg) + GTP (37.5 mg/kg), (4) APAP (400 mg/kg) + GTP (75 mg/kg), and (5) APAP (400 mg/kg) + GTP (150 mg/kg). All animals were fasted overnight before APAP administration. A single dose of 400 mg/kg APAP was given by gavage. Different doses of GTP were given orally 4 h after the APAP administration and continuously given every 8 h until sacrificed. Mice were sacrificed at 4, 12, 20, and 48 h after the first treatment of GTP. Serum and liver samples were harvested for further analysis. For the survival rate study, different groups of mice were monitored for their survival within 52 hours after APAP overdose.

### 2.5. Histological and Biochemical Assessments

Mouse liver tissues were harvested and immediately fixed in 4% methyl aldehyde, embedded in paraffin, cut into sections, and stained with hematoxylin and eosin (H&E) following a standard protocol. H&E-stained liver sections were used to evaluate liver damage using a Nikon digital sight DS-FI2 (Nikon, Japan). Moreover, Pannoramic slice scanner with CaseViewer 2.2 scanning and browsing software was used to select the equal target area of the slices. After imaging, Image-Pro Plus 6.0 software was used to measure the necrotic tissue area of 6 visual fields of each slice and the corresponding area of whole tissue in each slice. The percentage of necrotic tissue area was then calculated.

Serum alanine aminotransferase (ALT) and aspartate aminotransferase (AST) activities and the levels of total liver GSH were measured with a commercial reagent kit (Nanjing Jiancheng Bioengineering Institute, Nanjing, China) according to the manufacturer's instruction.

### 2.6. Western Blot Analysis

Protein extracted from liver tissues was prepared using NP-40 lysis buffer (1% Tris-HCL, 137 mm NaCl, 10% glycerol, 1% NP-40, 2 mm EDTA, and 1% proteinase inhibitor). Protein samples from the same group of mice were mixed, and concentrations were determined by bicinchoninic acid (BCA) protein assay (Thermo Scientific, Rockford, Illinois). Protein extract was analyzed triplicately by Wes (ProteinSimple, San Jose, USA) to determine the expression of cyp2e1, cyp3a11, cyp1a6, and Mrp2. Wes is a novel methodology to determine protein expression. However, it was difficult to optimize the method to obtain satisfying results in determining the expression of several proteins in our study.

Therefore, additionally, protein samples were duplicately separated on 8%–15% sodium dodecyl sulfate polyacrylamide gel electrophoresis (SDS-PAGE) and electrophoretically transferred onto polyvinylidene fluoride membranes (Millipore, Bedford). The membranes were probed with primary antibody including cyp1a2, sult1a1, and P-gp. Immunodetection was performed using an electrochemiluminescence (ECL) detection kit (Engreen Biosystem, Beijing, China) and analyzed using Quantity One software (Bio-Rad Laboratories, Hercules).

### 2.7. Statistical Analysis

Data were expressed as mean ± standard error of the mean (SEM). One-way ANOVA followed by the unpaired Student's *t*-test or Dunnett's multiple comparison post hoc test was performed using SPSS 17.0. *P* < 0.05 was considered statistically significant.

## 3. Results

### 3.1. Determination of Green Tea Polyphenol Extract

**Figure 1:**
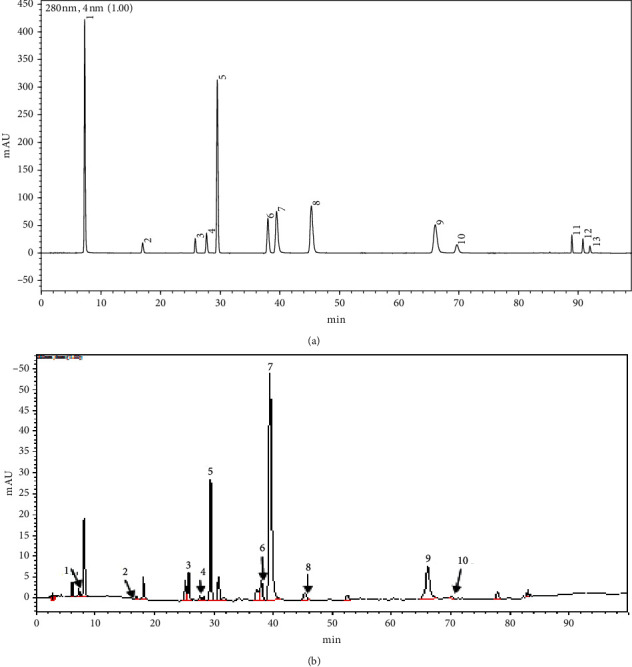
Representative HPLC chromatogram of (a) mixed standard solution and (b) longjing GTP extract. The numbers of the peaks in this figure coincide with the compound numbers in Table 1.

Different components in GTP extract were determined and quantified with HPLC (Figure 1 and Table 1). Compared with the chromatogram of mixed standard solution, longjing GTP extract mainly consisted EGCG (288.83 *μ*g/mg, 23.18%), EGC (57.74 *μ*g/mg, 34.26%), and ECG (42.08 *μ*g/mg, 19.09%). However, TF, TF-3-G, and TF-3′-G were not detected.

### 3.2. Protective Effect of GTP against APAP-Induced Liver Injury in Mice

**Figure 2:**
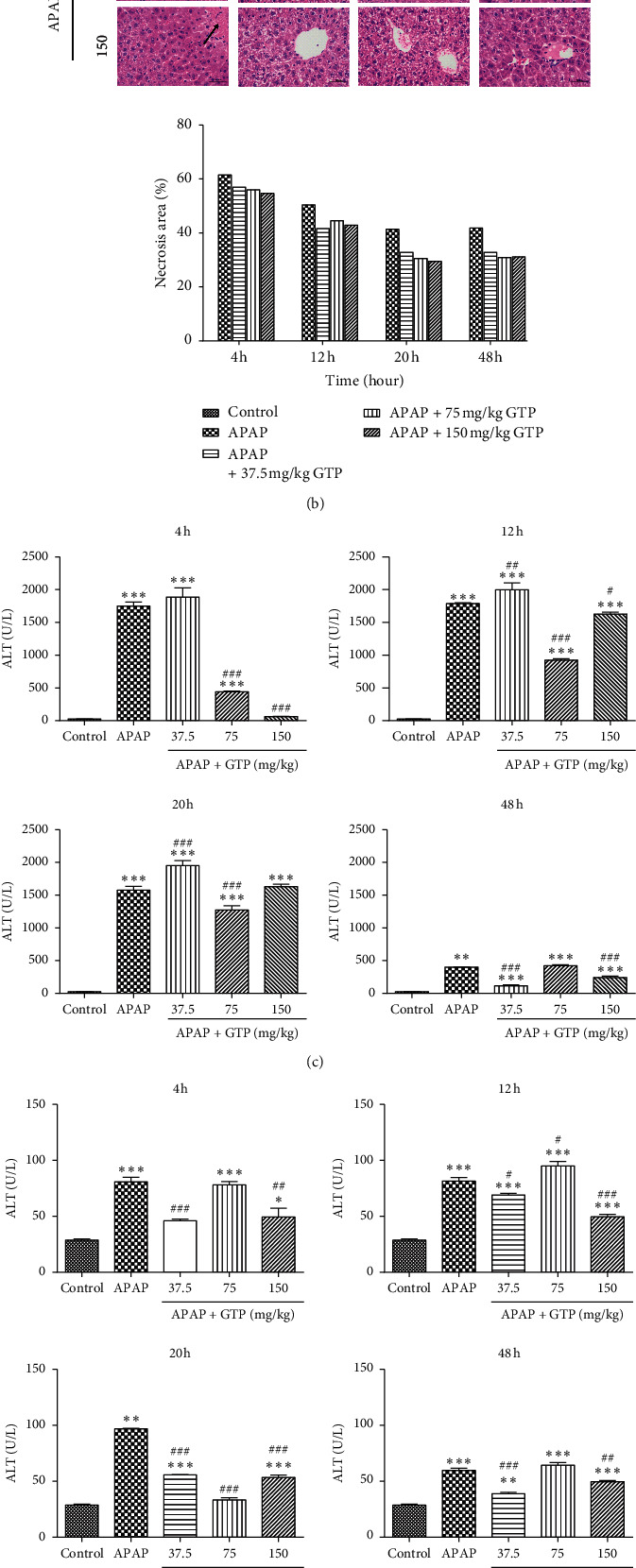
Protective effect of GTP against APAP-induced hepatotoxicity in mice. Different doses (37.5 mg/kg, 75 mg/kg, or 150 mg/kg) of GTP were orally given to mice 4 h after a single dose of APAP (400 mg/kg) administration and continuously given every 8 h afterward. (a) Survival rate was monitored within 52 h after APAP overdose (*n* = 10). Additionally, mice were sacrificed at 4, 12, 20, or 48 h after the first dose of GTP. Serum and liver samples were harvested and processed as indicated in Materials and Methods. (b) Histopathological analysis of mouse liver samples following H&E staining. (c) Serum ALT activity, (d) serum AST activity, and (e) total liver GSH levels from mice in each group (*n* = 10). Results are presented as mean ± SEM (*n* = 10). ^*∗*^*P* < 0.05,^*∗∗*^*P* < 0.01, and ^*∗∗∗*^*P* < 0.001 versus the control group. ^#^*P* < 0.05,^##^*P* < 0.01, and ^###^*P* < 0.001 versus the APAP group.

The survival of mice was monitored for a total of 52 h following the initial administration of APAP to evaluate the protective effect of GTP against APAP overdose. As shown in Figure 2(a), the survival rate of mice dropped to 70% after 52 h in the APAP overdose group when compared to the control group. However, different doses of GTP significantly increased the survival rate of mice in a dose-dependent manner (Figure 2(a)).

Histopathological analysis of H&E-stained liver sections indicated massive hepatotoxicity in mice treated with 400 mg/kg APAP for 4 h (Figure 2(b)). Large area of hepatocellular necrosis (50∼60%) was evident at 4 hours after APAP treatment. However, much less hepatocellular injury and necrosis was observed in liver sections of mice treated with different doses of GTP after APAP overdose in a dose-dependent manner.

APAP toxicity was also indicated by elevated serum ALT and AST levels after APAP overdose compared to the control group. Treatment with 75 mg/kg or 150 mg/kg GTP significantly reduced the ALT level at 4 h and 12 h time points. ALT activity was also lowered at 20 h time point by 75 mg/kg of GTP and at 48 h time point by 37.5 mg/kg or 150 mg/kg of GTP (Figure 2(c)). Similarly, all doses of GTP significantly reduced the AST levels at 20 h time point, while 37.5 mg/kg and 150 mg/kg of GTP reduced the AST levels at 4, 12, and 48 h time points (Figure 2(d)). Moreover, APAP treatment decreased the total GSH levels, which were reversed by different doses of GTP at 4 h time point, 37.5 mg/kg at 12 h time point, 75 mg/kg and 150 mg/kg at 20 h time point, and 150 mg/kg at 48 h time point (Figure 2(e)). Taken together, the results indicated that GTP exerted the protective effect against APAP-induced liver injury.

### 3.3. Effects of GTP on Drug Transporters in Mice

**Figure 3:**
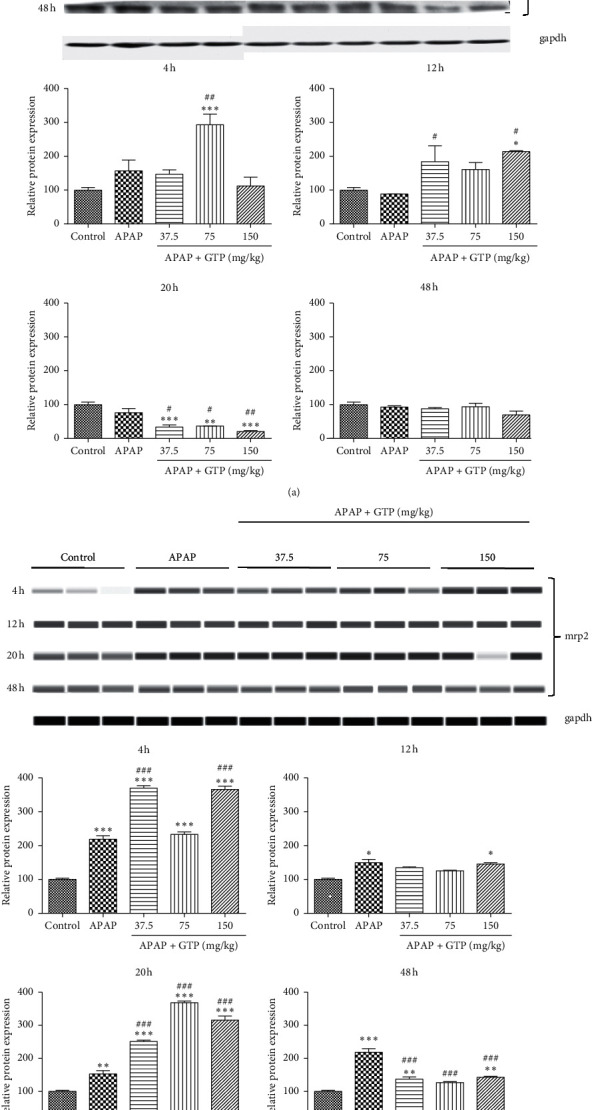
Effects of GTP on drug transporters in mice. Different doses (37.5 mg/kg, 75 mg/kg, or 150 mg/kg) of GTP were orally given to mice 4 h after a single dose of APAP (400 mg/kg) administration with an interval of 8 h. Mice were sacrificed at 4, 12, 20, or 48 h after the first dose of GTP. Liver samples were harvested and processed as indicated in Materials and Methods. P-gp (a) or Mrp2 (b) protein levels were analyzed. Data are expressed as fold change over the control group. Results are presented as mean ± SEM (*n* = 10). ^*∗*^*P* < 0.05,^*∗∗*^*P* < 0.01, and ^*∗∗∗*^*P* < 0.001 versus the control group. ^#^*P* < 0.05,^##^*P* < 0.01, and ^###^*P* < 0.001 versus the APAP group.

P-gp and Mrp2 are important transporters responsible for the efflux of diverse drugs from hepatocytes and therefore play a key role in detoxification. No effect was observed on P-gp expression with APAP overdose. Treatment of 75 mg/kg GTP markedly increased the P-gp expression at 4 h time point when compared to the control and APAP group. After 12 hours of APAP overdose, treatment with 37.5 mg/kg or 150 mg/kg GTP also increased the P-gp expression, which was repressed by all three doses of GTP at 20 h time point. No change of P-gp expression was detected after 48 h (Figure 3(a)). As for Mrp2, APAP overdose significantly induced the protein expression, which was further induced by 37.5 mg/kg and 150 mg/kg GTP after 4 h. The APAP-induced Mrp2 expression was upregulated by different doses of GTP after 20 h, but downregulated after 48 h (Figure 3(b)). Collectively, the effect of GTP on drug transporters such as P-gp and Mrp2 expression was implicated to be involved in the APAP biotransformation by inducing the expression at the early time points and reducing at the later time points, which was responsible for the excretion of toxic APAP metabolites NAPQI from the hepatocytes.

### 3.4. Effects of GTP on Phase-Metabolizing Enzymes in Mice

**Figure 4:**
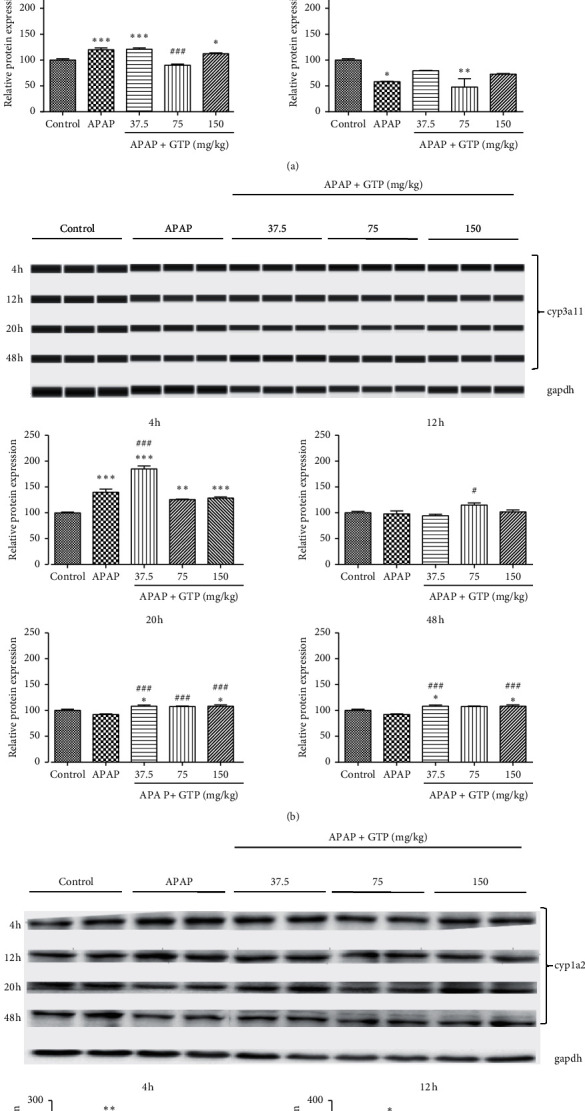
Effects of GTP on phase I metabolizing enzymes in mice. Different doses (37.5 mg/kg, 75 mg/kg, or 150 mg/kg) of GTP were orally given to mice 4 h after a single dose of APAP (400 mg/kg) administration with an interval of 8 h. Mice were sacrificed at 4, 12, 20, or 48 h after the first dose of GTP. Liver samples were harvested and processed as indicated in Materials and Methods. Cyp2e1 (a), cyp3a11 (b), or cyp1a2 (c) protein levels were analyzed. Data are expressed as fold change over the control group. Results are presented as mean ± SEM (*n* = 10). ^*∗*^*P* < 0.05,^*∗∗*^*P* < 0.01, and ^*∗∗∗*^*P* < 0.001 versus the control group. ^#^*P* < 0.05,^##^*P* < 0.01, and ^###^*P* < 0.001 versus the APAP group.

APAP was converted to NAPQI by multiple drug-metabolizing enzymes to initiate the APAP-induced hepatotoxicity. Therefore, the effect of GTP on the phase I enzyme expressions of cyp2e1, cyp3a11, and cyp1a2 was investigated. As shown in Figure 4(a), the protein expression of cyp2e1 was induced 4 h after APAP overdose, which was reduced by treatment with 75 mg/kg and 150 mg/kg GTP. The APAP-induced expression of cyp2e1 at 20 h time point was also reduced by 75 mg/kg GTP. As for cyp3a11, the protein expression was significantly induced 4 h after APAP overdose. GTP (37.5 mg/kg) further increased the expression of cyp3a11. Different doses of GTP also increased the expression of cyp3a11 at later time points (Figure 4(b)). No significant effect was observed on the expression of cyp1a2 following GTP treatment when compared to the APAP overdose group except for the 75 mg/kg GTP group after 20 h (Figure 4(c)). Collectively, these data indicated that the induction of cyp2e1 and cyp3a11 protein expression by GTP was involved in APAP biotransformation.

### 3.5. Effects of GTP on Phase II-Metabolizing Enzymes in Mice

**Figure 5:**
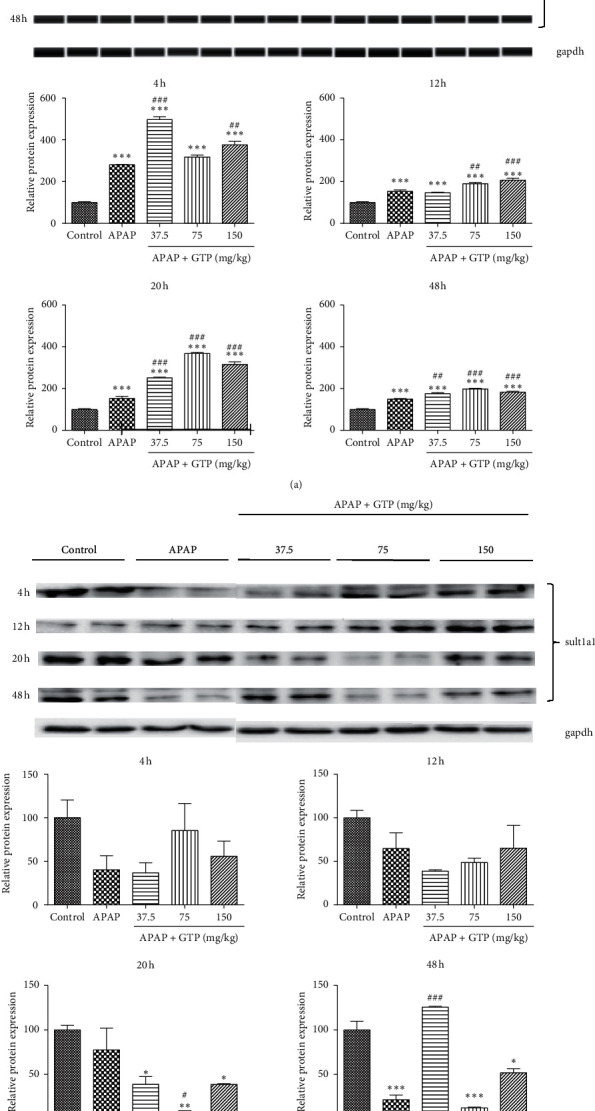
Effects of GTP on phase II metabolizing enzymes in mice. Different doses (37.5 mg/kg, 75 mg/kg, or 150 mg/kg) of GTP were orally given to mice 4 h after a single dose of APAP (400 mg/kg) administration with an interval of 8 h Mice were sacrificed at 4, 12, 20, or 48 h after the first dose of GTP. Liver samples were harvested and processed as indicated in Materials and Methods. Ugt1a6 (a) or sult1a1 (b) protein levels were analyzed. Data are expressed as fold change over the control group. Results are presented as mean ± SEM (*n* = 10). ^*∗*^*P* < 0.05,^*∗∗*^*P* < 0.01, and ^*∗∗∗*^*P* < 0.001 versus the control group. ^#^*P* < 0.05,^##^*P* < 0.01, and ^###^*P* < 0.001 versus the APAP group.

The majority of APAP was conjugated and converted into nontoxic metabolites by phase II drug-metabolizing enzymes. Therefore, the effects of GTP on ugt1a6 and sult1a1 were also investigated. Compared with the control group, APAP overdose significantly increased the ugt1a6 protein expression, which was further increased by the administration of different doses of GTP (Figure 5(a)). On the contrary, APAP or GTP had no effect on the protein expression of sult1a1 after 4 h and 12 h. However, all three doses of GTP lowered the expression of sult1a1 after 20 h when compared with the control group. Interestingly, APAP overdose markedly reduced the sult1a1 protein expression, which was dramatically increased by 37.5 mg/kg GTP after 48 h (Figure 5(b)). These data also suggested that the effect of GTP on ugt1a6 and sult1a1 expression was involved in the APAP biotransformation, with the induction of ugt1a6 expression increasing the conversion of APAP into nontoxic metabolites.

## 4. Discussion

Green tea has historically been one of the most commonly consumed beverages in a number of East Asian countries and also gained its popularity worldwide for decades. Polyphenols constitute approximately 30% of the dry weight of green tea, among which the majority are monomeric flavanols known as catechins [9]. The major catechins identified in green tea include catechin (C), epicatechin (EC), epigallocatechin (EGC), epicatechin-3-gallate (ECG), gallocatechin-3-gallate (GCG), and epigallocatechin-3-gallate (EGCG). Accumulating evidence has suggested that green tea polyphenols exhibit beneficial effects against a variety of diseases. For instance, targeting glycolysis with EGCG enhances the efficacy of chemotherapeutics in pancreatic cancer cells and xenografts [10]. Green tea polyphenols also have the potential to improve skeletal muscle metabolism in obese mice by improving glucose homeostasis, reducing lipid peroxidation, and increasing rate limiting enzymes of oxidative phosphorylation [11]. Moreover, EGCG exerts a neurorescue effect against functional and neurochemical deficits through regulating the iron-export protein ferroportin in substantia nigra and reducing oxidative stress in mice [12]. The current study aimed to investigate the protective effect of GTP against APAP-induced liver injury and the possible underlying mechanisms involved.

APAP overdose is one of the most frequent causes of acute liver injury in many countries, which is responsible for a number of emergency department visits, hospitalizations, or even death with up to $1.06 billion total national bill per year in the US [13]. The mechanisms underlying APAP-induced liver injury have been extensively studied [14–16]. Under normal condition, the majority of APAP is primarily metabolized by phase II enzymes UGTs or SULTs and excreted into urine via the kidney. The remaining APAP undergoes metabolism by phase I enzymes, mainly cytochrome P450 CYP2E1 and CYP1A2 to form a toxic metabolite NAPQI. The highly reactive NAPQI conjugates with GSH and is excreted into the bile without liver injury. Following APAP overdose, however, the accumulated NAPQI depletes intracellular and mitochondrial GSH and then reacts and forms covalent bonds with intracellular proteins, resulting in hepatotoxicity and hepatocyte necrosis. In the current study, APAP overdose (400 mg/kg) caused severe hepatic toxicity in mice as characterized by hepatocellular necrosis and elevated levels of serum ALT/AST and liver GSH. Treatment of GTP alleviated the liver injury, suggesting the protective effect of GTP against APAP-induced hepatotoxicity.

Multiple underlying mechanisms have been proposed in regulating the APAP-induced liver injury, among which drug metabolizing enzymes are involved, including phase I and II enzymes CYP2E1, CYP3A, CYP1A2, UGT, and GST [17]. A previous study showed that the hepatic drug-metabolizing enzyme activity was reduced after APAP treatment, but EGCG had no significant effects on it [8]. In our study, however, the protein expression of APAP-induced drug transporters and metabolizing enzymes was mostly induced by GTP treatment, which was followed by reduction in drug transporters at the later time points. Different studies have also indicated the induction of drug transporters such as P-gp and Mrps after APAP overdose [3, 18]. EGCG had no effect on P-gp and Mrp2/3 protein expressions in the rat liver. In our study, similar induction of Mrp2 protein expression was also detected after APAP overdose, but not in P-gp protein expression. Treatment with GTP further increased the transporter expression at early time points for the excretion of toxic APAP metabolites NAPQI from the hepatocytes, but reduced afterward. The protein expression of APAP-induced drug metabolizing enzymes, on the other hand, was mostly induced by GTP treatment to further increase the conversion of APAP into nontoxic metabolites.

Several possible explanations were proposed to be responsible for the discrepancy between the previous and the present studies. (1) Species difference, in which rats or mice were employed, respectively. (2) Different administration route of APAP, in which intraperitoneal injection or oral administration was conducted, respectively. (3) Different drug components, in which EGCG or the green tea extract was given, respectively. (4) Different strategy for drug administration, in which pretreatment of EGCG before APAP overdose or giving GTP after APAP overdose was conducted, respectively. Therefore, the above factors might all contribute to the different outcomes between the previous and the present studies. Moreover, induction in CYP expression but inhibition in activity was observed after treatment with herbs such as St John's wort and *Schisandra sphenanthera*. Further studies thus should investigate the effect of GTP on enzyme and transporter activities [6, 19]. Diverse signaling pathways are involved in regulating APAP-induced liver injury. For example, dynamic and coordinated regulation of KEAP1-NRF2-ARE and p53/p21 signaling pathways has been shown to be associated with compensatory liver regeneration after APAP-induced acute liver injury [20]. It is also interesting to determine whether GTP regulates KEAP1-NRF2-ARE and p53/p21 signaling pathways to protect against APAP-induced liver injury.

## 5. Conclusions

Taken together, the current study demonstrated that GTP protects against APAP-induced liver injury, possibly through regulating drug-metabolizing enzymes and transporters after APAP overdose.

## Figures and Tables

**Table 1:** The GTP extract quantitatively analyzed by HPLC.

No.	Components	Amount (*μ*g/mg)
1	Gallic acid (GA)	1.37
2	Gall catechin (GC)	4.95
3	Epigallocatechin (EGC)	57.74
4	Catechin (C)	32.22
5	Caffeine (CAF)	21.69
6	Epicatechin (EC)	21.14
7	Epigallocatechin gallate; (EGCG)	288.83
8	Gallocatechin gallate (GCG)	9.47
9	Epicatechin gallate (ECG)	42.08
10	Catechin gallate (CG)	5.99
11	Theaflavin (TF)	N.D.
12	Theaflavin-3-gallate (TF-3-G)	N.D.
13	Theaflavin-3′-gallate (TF-3′-G)	N.D.

## Data Availability

The data used to support the findings of this study are included within the article.

## Abbreviations

GTP: Green tea polyphenols
APAP: Acetaminophen
OTC: Over-the-counter
UGTs: UDP-glucuronosyltransferases
SULTs: Sulfotransferases
NAPQI: N-acetyl-p-benzoquinone imine
GSH: Glutathione
P-gp: P-glycoprotein
Mrps: Multidrug resistance-associated proteins
GA: Gallic acid
GC: Gall catechin
EGC: Epigallocatechin
C: Catechin
CAF: Caffeine
EC: Epicatechin
EGCG: Epigallocatechin gallate
GCG: Gallocatechin gallate
ECG: Epicatechin gallate
CG: Catechin gallate
TF: Theaflavin
TF-3-G: Theaflavin-3-gallate
TF-3′-G: Theaflavin-3′-gallate
CYP: Cytochrome P450.
